# Clinicopathological Predictors of Pathological Complete Response in HER2-Positive Breast Cancer Treated with Pertuzumab-Based Neoadjuvant Therapy: A Multicenter Real-World Study

**DOI:** 10.3390/medicina62040763

**Published:** 2026-04-15

**Authors:** Fatih Yıldız, Berkan Karabuga, Salih Karatlı, Merve Yalınkılıç, İlknur Deliktas Onur, Ece Bilgic Koylu, Sıla Soylu Kocoğlu, Emrah Eraslan, Aysegül Ilhan Gülesen, Erkan Erdur, Ozgen Ahmet Yildirim, Fatih Gurler, Hatime Arzu Yasar, Ferit Aslan

**Affiliations:** 1 Department of Medical Oncology, Dr. Abdurrahman Yurtaslan Ankara Oncology Training and Research Hospital, Ankara 06200, Turkey; drbkarabuga@gmail.com (B.K.); merveyalinkilic@gmail.com (M.Y.); ilknurdeliktas382@gmail.com (İ.D.O.); dremraheraslan@gmail.com (E.E.); 2 Department of Medical Oncology, Ankara Etlik City Hospital, Ankara 06170, Turkey; karatlisalih@hotmail.com (S.K.); ayse_ilhan85@hotmail.com (A.I.G.); ozgenayildirim@gmail.com (O.A.Y.); 3 Department of Medical Oncology, Ankara University Faculty of Medicine, Ankara 06050, Turkey; ecebilgicmd@gmail.com (E.B.K.); arzuyasar@gmail.com (H.A.Y.); 4 Department of Medical Oncology, Gazi University Faculty of Medicine, Ankara 06560, Turkey; drsilasoylu@gmail.com (S.S.K.); drfatihgurler@gmail.com (F.G.); 5 Department of Internal Medicine, Division of Medical Oncology, Gazi Yaşargil Training and Research Hospital, Diyarbakir 21070, Turkey; ererdur@hotmail.com; 6 Medical Oncology, Yüksek İhtisas University Affiliated Medicalpark Ankara Batıkent Hospital, Ankara 06370, Turkey; feritferhat21@gmail.com

**Keywords:** HER2-positive breast cancer, neoadjuvant therapy, pertuzumab, pathological complete response, systemic inflammatory biomarkers

## Abstract

*Background and Objectives*: Neoadjuvant systemic therapy incorporating dual HER2 blockade has significantly improved outcomes in patients with HER2-positive breast cancer. Pathological complete response (pCR) is an important surrogate endpoint associated with improved long-term survival. However, substantial heterogeneity in treatment response persists, and identifying factors associated with pCR remains clinically relevant. In addition to established clinicopathological variables, systemic inflammation-based biomarkers have recently been investigated as potential predictors of treatment response. *Materials and Methods*: In this multicenter retrospective study, we evaluated patients with stage II–III HER2-positive breast cancer who received pertuzumab-based neoadjuvant therapy followed by surgery between January 2023 and June 2025 across six oncology centers in Türkiye. Clinicopathological characteristics, treatment-related variables, and baseline systemic inflammation-based biomarkers were analyzed. Logistic regression analyses were performed to identify factors associated with pCR. *Results*: A total of 372 patients were included, and the overall pCR rate was 61%. Higher pCR rates were observed in patients with hormone receptor-negative tumors (71.4% vs. 54.3%, *p* = 0.001) and in premenopausal patients (68.7% vs. 53.4%, *p* = 0.003). In multivariate analysis, hormone receptor status (OR 2.25, 95% CI 1.41–3.60, *p* < 0.001), menopausal status (OR 1.90, 95% CI 1.22–2.94, *p* = 0.005), neoadjuvant treatment regimen (OR 2.15, 95% CI 1.05–4.41, *p* = 0.037), and perineural invasion (OR 2.61, 95% CI 1.10–6.22, *p* = 0.030) were independently associated with pCR. In contrast, systemic inflammation-based biomarkers did not demonstrate significant associations with pCR, and ROC analyses showed limited discriminatory ability (AUC values approximately 0.5). *Conclusions*: In patients with HER2-positive breast cancer treated with pertuzumab-based neoadjuvant therapy, treatment response appears to be primarily influenced by clinicopathological and treatment-related factors rather than systemic inflammatory status. Peripheral blood inflammatory biomarkers derived from routine laboratory parameters showed limited value in predicting pCR in this setting.

## 1. Introduction

Breast cancer is the most frequently diagnosed malignancy among women worldwide and remains a major cause of cancer-related mortality, with more than 2.3 million new cases and over 670,000 deaths annually according to recent global estimates [1]. It represents a biologically heterogeneous disease composed of distinct molecular subtypes with different prognostic and therapeutic implications [2]. Classification based on hormone receptor (HR) expression and human epidermal growth factor receptor 2 (HER2) status remains central to clinical decision-making [2]. Approximately 15–20% of breast cancers exhibit HER2 overexpression or ERBB2 gene amplification, a subtype historically associated with aggressive tumor behavior prior to the development of HER2-targeted therapies [3]. The introduction of HER2-directed treatment strategies has markedly improved outcomes and has substantially altered the natural history of HER2-positive breast cancer [4].

Neoadjuvant systemic therapy has become a standard treatment approach in patients with HER2-positive early and locally advanced breast cancer, as it enables both tumor downstaging and real-time assessment of treatment response [5]. In this setting, pathological complete response (pCR), defined as the absence of invasive disease in the breast and axillary lymph nodes, has emerged as a clinically meaningful endpoint associated with improved long-term survival outcomes [5,6]. The therapeutic evolution in HER2-positive disease has been shaped by pivotal trials demonstrating the benefit of HER2-targeted therapy, beginning with trastuzumab-based regimens and subsequently advancing to dual HER2 blockade with pertuzumab, which has consistently resulted in higher pCR rates in studies such as NeoSphere and TRYPHAENA [7,8,9]. Further optimization of treatment strategies has been achieved with trials such as TRAIN-2 [10]. More recently, antibody–drug conjugates have been introduced into earlier disease settings, and neoadjuvant strategies incorporating trastuzumab deruxtecan in combination with anti-HER2 therapy have demonstrated pCR rates approaching 60–70%, further underscoring the rapidly evolving treatment landscape in HER2-positive breast cancer [11].

Despite these advances, a significant proportion of patients do not achieve pCR following neoadjuvant therapy, indicating substantial heterogeneity in treatment response [6]. Several clinicopathological factors have been associated with pCR in breast cancer, including hormone receptor status, tumor grade, proliferation index, and HER2 expression levels [6,12]. In particular, HR-negative tumors and biologically more proliferative subtypes tend to demonstrate higher pCR rates, whereas HR-positive tumors often show relative resistance to neoadjuvant therapy [6,12]. In addition to tumor-intrinsic features, increasing evidence suggests that the tumor microenvironment and host immune response play a critical role in modulating treatment sensitivity [13]. Tumor-infiltrating lymphocytes have been associated with improved response to neoadjuvant therapy and favorable prognosis, highlighting the importance of immune-related factors in determining therapeutic outcomes [13,14]. Moreover, pathological features such as lymphovascular invasion (LVI) and perineural invasion (PNI) have been associated with more aggressive tumor biology and poorer outcomes across multiple solid malignancies, and emerging evidence suggests that these features may also reflect treatment resistance and lower likelihood of achieving pCR in breast cancer [15,16].

Systemic inflammatory markers have emerged as potential surrogate indicators of the interaction between tumor biology and host immune response across multiple solid tumors [17]. Indices such as neutrophil-to-lymphocyte ratio, platelet-to-lymphocyte ratio, systemic immune-inflammation index (SII), and prognostic nutritional index (PNI) have been associated with treatment response, recurrence, and survival in various malignancies [17,18]. In gastrointestinal cancers, these markers have been shown to correlate with both treatment response and disease recurrence, supporting their role as clinically relevant biomarkers reflecting systemic immune and inflammatory status [19,20]. In breast cancer, although several studies have investigated the prognostic and predictive value of inflammatory indices, the results remain inconsistent, and there is no consensus regarding optimal cut-off values or their clinical applicability [21,22,23]. Furthermore, data specifically evaluating these markers in patients receiving contemporary neoadjuvant dual HER2 blockade are limited.

Therefore, the present study was designed to evaluate pathological complete response rates and to investigate factors associated with pCR in patients with HER2-positive breast cancer treated with neoadjuvant pertuzumab-based therapy. In addition to established clinicopathological variables, we aimed to assess the potential predictive value of systemic inflammatory markers, including SII and related indices, in order to better understand their role in treatment response and to address an important gap in the current literature.

## 2. Materials and Methods

### 2.1. Study Design and Patient Selection

This study was designed as a retrospective multicenter analysis. Patients with HER2-positive stage II–III breast cancer who received pertuzumab-based neoadjuvant therapy and completed surgery between January 2023 and June 2025 were identified from institutional databases.

Ethical approval for the retrospective analysis of anonymized patient data was obtained from the Ankara Oncology Hospital Local Ethics Committee (Approval date: 4 September 2025; Approval number: 2025-09/106). The study was conducted in accordance with the Declaration of Helsinki. Due to the retrospective design of the study, the requirement for informed consent was waived.

Patients were eligible for inclusion if they met the following criteria: histologically confirmed invasive breast carcinoma; HER2-positive disease (HER2 score 3+ by immunohistochemistry or HER2 amplification confirmed by in situ hybridization in patients with HER2 score 2+); clinical stage II–III disease at the time of diagnosis; receipt of pertuzumab-based neoadjuvant systemic therapy followed by definitive breast surgery; and availability of complete clinicopathological data and baseline laboratory parameters prior to treatment initiation.

Patients were excluded if they had metastatic disease at the time of diagnosis, prior systemic therapy or radiotherapy for breast cancer, active infection, inflammatory disease, or hematologic disorders that could affect blood cell counts, missing baseline laboratory parameters required for the calculation of inflammatory indices, incomplete pathological response data after surgery, ongoing neoadjuvant therapy at the time of analysis, neoadjuvant treatment regimens not including pertuzumab, or absence of definitive surgical treatment following neoadjuvant therapy.

Clinicopathological characteristics including menopausal status, tumor stage, hormone receptor status, HER2 score, tumor grade, lymphovascular invasion (LVI), perineural invasion (PNI), and treatment-related variables were retrieved from institutional medical records.

The primary endpoint of the study was pathological complete response (pCR) in both the breast and axilla. pCR was defined as the absence of residual invasive tumor in the breast and axillary lymph nodes following neoadjuvant therapy (ypT0/is ypN0). The duration of neoadjuvant therapy was defined as the interval between the first and the last cycle of neoadjuvant treatment.

### 2.2. Systemic Inflammation-Based Biomarkers

Systemic inflammation-based biomarkers were calculated using baseline complete blood count parameters obtained prior to treatment initiation. Absolute neutrophil, lymphocyte, monocyte, and platelet counts were expressed as ×10^9^/L, while hemoglobin and albumin levels were expressed in g/L.

The neutrophil-to-lymphocyte ratio (NLR) was calculated as the absolute neutrophil count divided by the absolute lymphocyte count. The platelet-to-lymphocyte ratio (PLR) was defined as the absolute platelet count divided by the absolute lymphocyte count. The monocyte-to-lymphocyte ratio (MLR) was calculated as the absolute monocyte count divided by the absolute lymphocyte count. The neutrophil-to-monocyte ratio (NMR) was calculated as the absolute neutrophil count divided by the absolute monocyte count. The lymphocyte-to-monocyte ratio (LMR) was calculated as the absolute lymphocyte count divided by the absolute monocyte count.

The systemic immune-inflammation index (SII) was calculated as platelet count × neutrophil count/lymphocyte count. The systemic inflammation response index (SIRI) was calculated as neutrophil count × monocyte count/lymphocyte count. The pan-immune-inflammation value (PIV) was calculated as neutrophil count × platelet count × monocyte count/lymphocyte count. The hemoglobin, albumin, lymphocyte, and platelet (HALP) score was calculated using the following formula: hemoglobin × albumin × lymphocyte count/platelet count.

Baseline blood samples used for the calculation of inflammatory indices were obtained during routine clinical evaluation prior to treatment initiation and were collected within 7 days before the first cycle of neoadjuvant therapy.

Because there are no universally accepted or validated cut-off values for systemic inflammatory biomarkers in patients with HER2-positive breast cancer treated with neoadjuvant pertuzumab-based therapy, all inflammatory indices were primarily analyzed as continuous variables. This approach was preferred to avoid arbitrary categorization and potential loss of statistical power. Although various cut-off values have been proposed in the literature, these thresholds are highly heterogeneous and not standardized across studies or disease settings. Therefore, no predefined cut-off values were used in the primary analyses.

### 2.3. Statistical Analysis

Associations between clinicopathological characteristics and systemic inflammation-based biomarkers and pathological complete response (pCR) were evaluated using the chi-square test for categorical variables and the independent samples Student’s t-test for continuous variables. Continuous variables were expressed as mean ± standard deviation (SD) or median (interquartile range), while categorical variables were presented as frequencies and percentages. The normality of continuous variables was assessed using the Shapiro–Wilk test and visual inspection of histograms.

Clinicopathological variables were initially assessed using univariate logistic regression analysis to identify factors associated with achieving pCR. Systemic inflammation-based biomarkers were evaluated separately using ROC curve analysis to explore their potential predictive value. Variables showing statistical significance in univariate analyses were subsequently included in multivariate logistic regression models to determine independent predictors of pCR. The results of logistic regression analyses were reported as odds ratios (ORs) with 95% confidence intervals (CIs). Variables with *p* < 0.10 in univariate analysis were considered eligible for inclusion in the multivariate model. The calibration of the multivariate model was assessed using the Hosmer–Lemeshow goodness-of-fit test, and the explanatory power of the model was evaluated using Nagelkerke R^2^.

The predictive performance of systemic inflammation-based biomarkers for pCR was further evaluated using receiver operating characteristic (ROC) curve analysis, and the area under the curve (AUC) was calculated to assess their discriminatory ability. ROC analyses were considered exploratory and were not used to define definitive cut-off values.

All statistical analyses were performed using IBM SPSS Statistics version 27 (IBM Corp., Armonk, NY, USA). A two-sided *p* value < 0.05 was considered statistically significant.

## 3. Results

### 3.1. Patient Characteristics

A total of 372 patients with stage II-III HER2-positive breast cancer who received pertuzumab-based neoadjuvant systemic therapy were included in the study. The mean age of the patients was 49.7 ± 11.9 years. Overall, pathological complete response (pCR) was achieved in 61% of patients following neoadjuvant treatment.

The baseline clinicopathological characteristics of the study population are summarized in Table 1. Nearly half of the patients were premenopausal (48.1%), while 51.9% were postmenopausal. At diagnosis, most tumors were T2 stage (66.1%), followed by T1 (18.3%), T3 (8.3%), and T4 (7.3%). Axillary lymph node involvement was present in 90.6% of patients.

The most common histological subtype was invasive ductal carcinoma (74.7%). Hormone receptor-positive disease was observed in 62.4% of patients, while 37.6% had hormone receptor-negative tumors. The majority of tumors were high grade, with 62.4% classified as grade 3. Lymphovascular invasion (LVI) and perineural invasion (PNI) were present in 10.8% and 6.5% of patients, respectively.

### 3.2. Neoadjuvant Treatment Characteristics

The median duration of neoadjuvant treatment was 4.9 months (range, 1.8–17.1). Most patients received an anthracycline-based regimen (89.8%). The most commonly administered treatment protocol was four cycles of doxorubicin and cyclophosphamide followed by docetaxel combined with trastuzumab and pertuzumab (4AC + 4DTP), which was administered to 82.5% of patients.

An additional 7.3% of patients received 4AC followed by weekly paclitaxel with trastuzumab and pertuzumab, while 10.2% were treated with the TCHP regimen. Overall, 94.1% of patients completed neoadjuvant treatment, and the majority received four cycles of pertuzumab (91.7%) (Table 2).

### 3.3. Clinicopathological Factors Associated with pCR

pCR rates differed significantly across several clinicopathological subgroups (Table 3). Patients with hormone receptor-negative tumors achieved significantly higher pCR rates compared with those with hormone receptor-positive disease (71.4% vs. 54.3%, *p* = 0.001). Similarly, ER-negative and PR-negative tumors were associated with higher pCR rates (*p* = 0.008 and *p* = 0.012, respectively).

Premenopausal patients were more likely to achieve pCR compared with postmenopausal patients (68.7% vs. 53.4%, *p* = 0.003). In addition, patients with HER2 Score 3+ tumors had significantly higher pCR rates than those with HER2 Score 2+ disease (62.7% vs. 45.2%, *p* = 0.029).

Neoadjuvant treatment regimen was also associated with treatment response, with higher pCR rates observed in patients receiving anthracycline-based regimens compared with TCHP (62.6% vs. 44.7%, *p* = 0.033).

Among pathological factors, PNI status was significantly associated with pCR, with lower response rates observed in patients with PNI-positive tumors (41.7% vs. 62.1%, *p* = 0.048).

### 3.4. Logistic Regression Analysis

In univariate logistic regression analysis, menopausal status, hormone receptor status, HER2 score, neoadjuvant treatment regimen, and PNI status were associated with pCR.

In the multivariate logistic regression model, hormone receptor status, menopausal status, neoadjuvant treatment regimen, and PNI status remained independently associated with pCR (Table 4).

Hormone receptor-negative disease was independently associated with a higher likelihood of achieving pCR (OR 2.25, 95% CI 1.41–3.60, *p* < 0.001). Premenopausal status was also an independent predictor of pCR (OR 1.90, 95% CI 1.22–2.94, *p* = 0.005).

Neoadjuvant treatment regimen remained significantly associated with treatment response (OR 2.15, 95% CI 1.05–4.41, *p* = 0.037). In addition, PNI status emerged as an independent factor, with PNI-negative patients having a higher likelihood of achieving pCR (OR 2.61, 95% CI 1.10–6.22, *p* = 0.030).

HER2 score did not retain statistical significance in the multivariate model (*p* = 0.102).

### 3.5. Systemic Inflammation-Based Biomarkers

No significant differences were observed between patients who achieved pCR and those with residual disease in terms of baseline systemic inflammatory indices, including NLR, PLR, MLR, NMR, LMR, SII, SIRI, HALP, and PIV (all *p* > 0.05).

**Figure 1:**
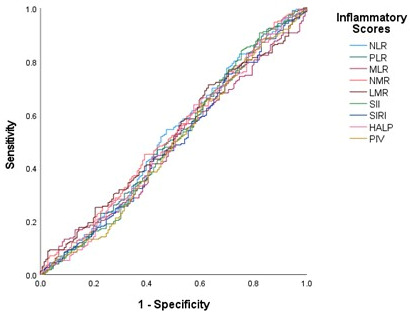
ROC curves of inflammatory biomarkers for predicting pCR. NLR–neutrophil-to-lymphocyte ratio; PLR–platelet-to-lymphocyte ratio; MLR–monocyte-to-lymphocyte ratio; NMR–neutrophil-to-monocyte ratio; LMR–lymphocyte-to-monocyte ratio; SII–systemic immune-inflammation index; SIRI–systemic inflammation response index; HALP–hemoglobin–albumin–lymphocyte–platelet score; PIV–pan-immune-inflammation value.

Receiver operating characteristic (ROC) curve analysis demonstrated that none of the investigated inflammatory indices had meaningful discriminatory ability for predicting pCR, with all AUC values close to 0.5 (Figure 1 and Table 5). These findings suggest that systemic inflammation-based biomarkers have limited predictive value for pCR in this patient population.

## 4. Discussion

In this multicenter retrospective study, we evaluated real-world pathological complete response (pCR) rates and investigated clinicopathological and systemic inflammation-based predictors of treatment response in patients with stage II-III HER2-positive breast cancer treated with pertuzumab-based neoadjuvant therapy. The overall pCR rate in our cohort was 61%, which is consistent with rates reported in pivotal clinical trials evaluating dual HER2 blockade in the neoadjuvant setting [7,8]. In addition, we identified hormone receptor status, menopausal status, neoadjuvant treatment regimen, and perineural invasion (PNI) as independent predictors of pCR, whereas systemic inflammation-based biomarkers did not demonstrate predictive value. These findings highlight the dominant role of tumor biology and treatment-related factors over systemic inflammatory status in determining treatment response in this setting.

The introduction of dual HER2 blockade has substantially improved treatment outcomes in HER2-positive breast cancer. In the phase II NeoSphere trial, the addition of pertuzumab to trastuzumab and docetaxel significantly increased pCR rates compared with trastuzumab-based therapy alone [7]. Similarly, the TRYPHAENA study demonstrated high pCR rates with pertuzumab-containing regimens while maintaining acceptable cardiac safety [8]. The pCR rate observed in our real-world cohort is comparable with these pivotal studies and further supports the effectiveness of pertuzumab-based neoadjuvant therapy in routine clinical practice [7,8,12,13,14].

More recent trials have further refined treatment strategies in HER2-positive disease. The KRISTINE trial demonstrated higher pCR rates in patients receiving chemotherapy combined with dual HER2 blockade compared with T-DM1-based therapy [24]. Likewise, the TRAIN-2 study reported comparable pCR rates between anthracycline-containing and non-anthracycline regimens in the presence of dual HER2 blockade, highlighting the central role of effective HER2 inhibition rather than chemotherapy backbone [10]. More recently, antibody–drug conjugates have been investigated in earlier disease settings. In the phase III DESTINY-Breast11 trial, neoadjuvant trastuzumab deruxtecan-based strategies achieved pCR rates exceeding 65% in selected patient populations, further expanding the therapeutic landscape of HER2-positive breast cancer [11].

Consistent with prior literature, we observed significantly higher pCR rates among patients with hormone receptor-negative tumors compared with those with hormone receptor-positive disease [5,6]. In addition to hormone receptor status, several biological and molecular features have been associated with the likelihood of achieving pCR or residual disease following neoadjuvant therapy in breast cancer. Increasing evidence suggests that intrinsic biological subtype, proliferation-related markers, and genomic features reflecting tumor aggressiveness may influence treatment sensitivity. Recent studies have further emphasized the importance of biological subtype in determining response patterns and residual disease burden following neoadjuvant therapy, highlighting the complex interaction between tumor biology and therapeutic response [25]. These observations support the concept that treatment response is largely driven by tumor-intrinsic biological characteristics rather than a single clinical or laboratory parameter. This finding reflects the biological heterogeneity within HER2-positive breast cancer, where HR-negative tumors are more dependent on HER2 signaling pathways and therefore exhibit greater sensitivity to HER2-targeted therapies.

Another notable finding of our study was the higher pCR rate observed among premenopausal patients. Although menopausal status itself may not directly determine treatment response, it likely reflects underlying tumor biology, as younger patients more frequently present with highly proliferative and biologically aggressive tumors [6].

We also observed that patients with HER2 IHC 3+ tumors achieved higher pCR rates compared with those with HER2 IHC 2+/ISH-positive disease. This observation supports the concept that the degree of HER2 protein overexpression may influence treatment sensitivity, potentially reflecting greater oncogenic dependence on HER2 signaling [3].

In our cohort, anthracycline-containing neoadjuvant regimens were associated with higher pCR rates compared with non-anthracycline regimens. However, this observation should be interpreted with considerable caution. Because treatment allocation was not randomized in this retrospective real-world cohort, regimen selection was likely influenced by several clinical and institutional factors, including tumor burden, nodal status, physician preference, patient comorbidities, and concerns regarding anthracycline-related toxicity. Consequently, the observed association may reflect treatment selection bias or residual confounding rather than a true superiority of anthracycline-based therapy. Importantly, randomized evidence from the TRAIN-2 trial has demonstrated comparable pCR rates between anthracycline-containing and non-anthracycline regimens when dual HER2 blockade is administered, supporting the interpretation that effective HER2-targeted therapy rather than chemotherapy backbone is the primary driver of treatment response [10].

Interestingly, PNI emerged as an independent factor associated with pCR in the multivariate model. However, this finding should be interpreted with caution. Only a small proportion of patients in our cohort were reported as PNI-positive, which may limit the stability of the observed association and result in wide confidence intervals. In addition, variability in pathological reporting across centers may have influenced the identification of PNI. While PNI has been recognized as an adverse prognostic feature in several solid tumors, it is not currently an established predictive biomarker of treatment response in breast cancer [16,26]. Therefore, this observation should be considered hypothesis-generating and warrants validation in larger prospective studies. Although variables such as clinical tumor stage, nodal status, tumor grade, and Ki-67 index are recognized determinants of treatment response in breast cancer, these variables were not significantly associated with pCR in the univariate analysis in our cohort and were therefore not included in the final multivariable model.

One of the primary objectives of our study was to evaluate the predictive value of systemic inflammation-based biomarkers. These markers were selected because they are inexpensive, readily available, and routinely obtained in daily clinical practice, making them attractive candidates for real-world risk stratification. In contrast to tissue-based biomarkers such as tumor-infiltrating lymphocytes (TILs), which require specialized pathological assessment and are not consistently reported across centers, systemic inflammatory indices can be easily integrated into routine clinical workflows.

In contrast to several reports in other solid tumors, we did not observe significant associations between inflammatory indices—including NLR, PLR, SII, SIRI, HALP, and PIV—and pCR. Previous meta-analyses and large cohort studies have suggested that systemic inflammatory markers, particularly NLR and SII, may be associated with prognosis and treatment response across multiple malignancies [17,18,21]. However, in breast cancer, several studies have reported limited or no predictive value of these markers in the neoadjuvant setting [22,23]. Several methodological factors may also have influenced the evaluation of inflammatory biomarkers in this study. First, systemic inflammatory indices were calculated using a single baseline measurement obtained prior to treatment initiation, and dynamic changes during therapy were not assessed. Emerging evidence suggests that longitudinal changes in inflammatory markers during treatment may provide additional prognostic or predictive information. Second, as this was a multicenter retrospective study, potential inter-center variability in laboratory methods, timing of blood sampling, and supportive medications may have introduced additional variability in these parameters. Therefore, although our findings suggest that baseline inflammatory indices were not useful predictors of pCR in this cohort, these results should be interpreted cautiously and do not necessarily exclude a potential role of systemic inflammatory status in treatment response.

Several factors may explain these findings. First, treatment response in HER2-positive breast cancer is largely driven by tumor-intrinsic molecular features and HER2 pathway dependency, which may outweigh the influence of systemic inflammatory status [6,12]. Second, peripheral blood-derived inflammatory markers may not accurately reflect the local tumor immune microenvironment, where tumor-infiltrating lymphocytes (TILs) have been consistently shown to predict response to HER2-targeted therapy and improve survival outcomes [27]. Third, the use of a single baseline measurement may not capture dynamic immune changes during treatment, limiting the ability of these markers to predict treatment response [22,23].

Our study has several strengths, including its multicenter design and relatively large sample size, providing robust real-world data on patients treated with contemporary pertuzumab-based neoadjuvant therapy. In addition, the inclusion of a comprehensive panel of systemic inflammation-based biomarkers allowed for a detailed evaluation of their predictive value within a single cohort.

However, several limitations should be considered. The retrospective design introduces the potential for selection bias and residual confounding. Treatment regimens were not randomized, which may have influenced the observed association between chemotherapy backbone and pCR. Additionally, inter-institutional variability in treatment approaches and pathological assessment may have contributed to heterogeneity in the data. Another important limitation is the lack of standardized tumor-infiltrating lymphocyte (TIL) data. In our cohort, TIL assessment was not routinely reported across participating centers, resulting in a high proportion of cases classified as unknown. Because TILs represent one of the most established immune-related biomarkers associated with response to HER2-targeted therapy, the absence of reliable TIL data limits our ability to interpret systemic inflammatory biomarkers within the broader context of tumor–immune interactions. Furthermore, the relatively small number of patients with PNI-positive disease may limit the robustness of this finding, and larger studies with adequate statistical power are needed for validation. Finally, systemic inflammatory markers were assessed at a single time point and may not reflect dynamic changes during treatment.

Despite these limitations, our study makes several important contributions to the literature. The role of biomarkers in the efficacy of dual HER2 blockade has mostly been investigated in clinical trials. Real-world experiences investigating the effect of biomarkers on pCR are quite limited. Our study represents a relatively large multicenter real-world analysis. Furthermore, our study evaluated a broad panel of systemic inflammatory biomarkers, revealing that these markers may have limited utility in predicting pCR in HER2-positive breast cancer. Importantly, our findings support the need to move beyond single-parameter biomarkers in predicting pCR towards integrated approaches that include tumor biology, immune microenvironment, and systemic host factors.

## 5. Conclusions

In conclusion, pertuzumab-based neoadjuvant therapy achieves high pCR rates in patients with stage II–III HER2-positive breast cancer in real-world clinical practice. In this multicenter cohort, clinicopathological factors such as hormone receptor status, menopausal status, treatment regimen, and perineural invasion were associated with treatment response. Although we evaluated a comprehensive panel of systemic inflammation-based biomarkers derived from routine complete blood count parameters, these markers did not demonstrate meaningful predictive value for pCR in our cohort. These findings suggest that treatment response in HER2-positive breast cancer treated with contemporary dual HER2 blockade may be more strongly influenced by tumor biology than by peripheral systemic inflammatory status. However, given the retrospective design and potential for residual confounding, these observations should be interpreted cautiously and considered hypothesis-generating, warranting validation in future prospective studies.

## Figures and Tables

**Table 1:** Demographic and clinicopathological characteristics of patients.

		Total *n* = 372 (%)
Menopausal status	Premenopausal	179 (48.1)
	Postmenopausal	193 (51.9)
T stage at the time of diagnosis	T1	68 (18.3)
	T2	246 (66.1)
	T3	31 (8.3)
	T4	27 (7.3)
BRCA status	Negative/unknown	366 (98.4)
	Positive	6 (1.6)
Axillary status at the time of diagnosis	Negative	35 (9.4)
	Positive	337 (90.6)
Histology	IDC	278 (74.7)
	ILC	9 (2.4)
	NOS/others	77 (20.7)
	Mix	8 (2.2)
HR status	Negative	140 (37.6)
	Positive	232 (62.4)
Age	<40	76 (20.4)
	40–50	116 (31.2)
	>50	180 (48.4)
HER2 score	Score 2+	42 (11.3)
	Score 3+	330 (88.7)
Tumor grade	Grade 1	1 (0.3)
	Grade 2	139 (37.4)
	Grade 3	232 (62.4)
LVI status	Negative/unknown	332 (89.2)
	Positive	40 (10.8)
PNI status	Negative/unknown	348 (93.5)
	Positive	24 (6.5)
Ki-67 index	<20	28 (7.5)
	20–50	200 (53.8)
	>50	144 (38.7)
TIL	Unknown/<10	362 (97.3)
	10–40	4 (1.1)
	40–50	3 (0.8)
	50–60 (LPBC)	3 (0.8)

BRCA—*BRCA* gene mutation (Breast Cancer gene 1/2); IDC—invasive ductal carcinoma; ILC—invasive lobular carcinoma; HR—hormone receptor; LVI— lymphovascular invasion; PNI—perineural invasion; TIL—tumor-infiltrating lymphocytes; NOS—not otherwise specified; LPBC—lymphocyte-predominant breast cancer.

**Table 2:** Neoadjuvant treatment characteristics of the study population.

		*n* = 372 (%)
Neoadjuvant treatment regimens	4AC + 4DTP	307 (82.5)
	4AC + 12w paclitaxel + PT	27 (7.3)
	TCHP	38 (10.2)
Neoadjuvant anthracycline status	No	38 (10.2)
	Yes	334 (89.8)
Neoadjuvant treatment completing status	No	22 (5.9)
	Yes	350 (94.1)
Neoadjuvant pertuzumab cycle	2–3 cycles	6 (1.6)
	4 cycles	341 (91.7)
	5–6 cycles	25 (6.7)

AC—doxorubicin-cyclophosphamide; DTP—docetaxel–trastuzumab–pertuzumab; PT—paclitaxel–trastuzumab; TCHP—docetaxel-carboplatin–trastuzumab–pertuzumab.

**Table 3:** Clinicopathological factors associated with pathological complete response (pCR).

		Total *n* (%)	pCR *n* (%)	*p*-Value
HR status	Negative	140 (37.6)	100 (71.4)	0.001 *
	Positive	232 (62.4)	126 (54.3)	
Neoadjuvant treatment regimen	AC+Tx+PT	334 (89.8)	209 (62.6)	0.033 *
	TCHP	38 (10.2)	17 (44.7)	
Menopausal status	Premenopausal	179 (48.1)	123 (68.7)	0.003 *
	Postmenopausal	193 (51.9)	103 (53.4)	
Ki-67 index	<20	28 (7.5)	21 (75.0)	0.076
	20–50	200 (53.8)	112 (56.0)	
	>50	144 (38.7)	93 (64.6)	
Age	<40	76 (20.4)	51 (67.1)	0.054
	40–50	116 (31.2)	77 (66.4)	
	>50	180 (48.4)	98 (54.4)	
Axillary status	Negative	35 (9.4)	25 (71.4)	0.205
	Positive	337 (90.6)	201 (59.6)	
T stage	T1	68 (18.3)	44 (64.7)	0.055
	T2	246 (66.1)	155 (63.0)	
	T3	31 (8.3)	12 (38.7)	
	T4	27 (7.3)	15 (55.6)	
HER2 score	Score 2+	42 (11.3)	19 (45.2)	0.029 *
	Score 3+	330 (88.7)	207 (62.7)	
PNI status	Negative	348 (93.5)	216 (62.1)	0.048 *
	Positive	24 (6.5)	10 (41.7)	

HR—hormone receptor; AC—doxorubicin plus cyclophosphamide; Tx—taxane; PT—paclitaxel–trastuzumab; PNI—perineural invasion; TCHP—docetaxel–carboplatin–trastuzumab–pertuzumab. * statistically significant.

**Table 4:** Logistic regression analysis of clinicopathological factors associated with pCR.

Univariate				Multivariate		
	OR	95% CI	*p*-Value	OR	95% CI	*p*-Value
Menopausal status	1.92	1.26–2.93	0.003 *	1.90	1.22–2.94	0.005 *
HR status	2.10	1.34–3.29	0.001 *	2.25	1.41–3.60	<0.001 *
HER2 status	2.03	1.07–3.89	0.030 *	1.76	0.90–3.50	0.102
Neoadjuvant treatment regimen	2.06	1.05–4.06	0.036 *	2.15	1.05–4.41	0.037 *
PNI status	2.29	0.99–5.30	0.053	2.61	1.10–6.22	0.030 *

HR—hormone receptor; PNI—perineural invasion; * statistically significant.

**Table 5:** ROC analysis of inflammatory indices for prediction of pCR.

	**AUC**	**95% CI**	***p*** **-Value**
NLR	0.516	0.45–0.58	0.61
PLR	0.504	0.44–0.57	0.90
MLR	0.484	0.42–0.54	0.60
NMR	0.523	0.46–0.58	0.61
LMR	0.516	0.46–0.58	0.61
SII	0.500	0.44–0.56	0.99
SIRI	0.488	0.43–0.56	0.69
HALP	0.500	0.44–0.56	0.99
PIV	0.483	0.42–0.55	0.58

NLR–neutrophil-to-lymphocyte ratio; PLR–platelet-to-lymphocyte ratio; MLR–monocyte-to-lymphocyte ratio; NMR–neutrophil-to-monocyte ratio; LMR–lymphocyte-to-monocyte ratio; SII–systemic immune-inflammation index; SIRI–systemic inflammation response index; HALP–hemoglobin–albumin–lymphocyte–platelet score; PIV–pan-immune-inflammation value.

## Data Availability

The original contributions presented in the study are included in the article; further inquiries can be directed to the corresponding author.
